# Digital health literacy and associated factors among community-dwelling older adults with multimorbidity: a cross-sectional study

**DOI:** 10.3389/fpubh.2026.1841486

**Published:** 2026-07-08

**Authors:** Qi Ao, Xin Luo, Zhe Zhang, Yuqing Wang, Peili Xu

**Affiliations:** 1 School of Nursing, Anhui University of Chinese Medicine, Anhui, China; 2 Nursing Department, The First People’s Hospital of Hefei, Anhui, China; 3 School of Nursing, Bengbu Medical University, Anhui, China

**Keywords:** community-dwelling population, digital health literacy, health activation, multimorbidity, older adults, social support, technophobia

## Abstract

**Background:**

Digital health technologies are increasingly embedded in chronic disease management, yet older adults with multimorbidity may have difficulty accessing, evaluating, and applying digital health information. This study assessed digital health literacy (DHL) and examined its sociodemographic, clinical, digital, and psychosocial correlates among community-dwelling older adults with multimorbidity.

**Methods:**

This community-based cross-sectional study included a convenience sample of 345 adults aged 60 years or older with at least two physician-diagnosed chronic conditions who were recruited from three community hospitals in Hefei, China, between November 2025 and February 2026. DHL was measured using the 15-item Digital Health Literacy Scale. Hierarchical linear regression evaluated associations with sociodemographic and clinical characteristics, Internet use, technology perceptions, health activation, technophobia, and reciprocal social support. Sensitivity and exploratory moderation analyses were also conducted.

**Results:**

The median DHL score was 39.00 (interquartile range, 27.00–55.00). In the fully adjusted model, older age was associated with lower DHL, whereas higher educational attainment, monthly income above 2,000 CNY, and longer daily Internet use were associated with higher DHL. Considering digital health information only slightly useful (B = −6.753; 95% CI, −9.144 to −4.363) and reporting difficulty using digital health tools (B = −3.855; 95% CI, −6.545 to −1.165) were associated with lower DHL. Higher health activation (B = 0.057; 95% CI, 0.013 to 0.100) and reciprocal social support (B = 0.253; 95% CI, 0.142 to 0.365) were associated with higher DHL, whereas technophobia was associated with lower DHL (B = −0.232; 95% CI, −0.327 to −0.138). The final model had an adjusted *R*^2^ of 0.877; strong intercorrelations and a maximum variance inflation factor of 5.929 indicated residual construct overlap. Sensitivity analyses preserved the direction of the psychosocial associations, and the technophobia-by-support interaction was not significant.

**Conclusion:**

DHL was modest and was associated with socioeconomic resources, digital exposure, technology perceptions, health activation, technophobia, and reciprocal social support. Interventions should combine accessible digital-skills training with efforts to improve usability, reduce technology-related fear, strengthen self-management capacity, and provide trusted human support. Because of the cross-sectional design and construct overlap, these findings should not be interpreted causally.

## 1 Introduction

Population ageing has become a major public health challenge worldwide. The World Health Organization reports that both the absolute number and proportion of older adults are increasing globally, and this demographic transition is occurring particularly rapidly in many middle-income countries (1). In China, population ageing is also accelerating. During the 14th Five-Year Plan period, China is projected to transition from a mildly aged society to a moderately aged society and is expected to enter a heavily aged stage around 2035 (2, 3). This demographic shift has placed increasing pressure on community-based healthcare systems, particularly in relation to long-term chronic disease management.

Multimorbidity, commonly defined as the coexistence of two or more chronic conditions in the same individual, is highly prevalent among older adults and represents a major challenge for primary care and community health services (4). Previous studies have reported that chronic diseases are common among community-dwelling older adults in China, with multimorbidity affecting a substantial proportion of this population (5, 6). Older adults with multimorbidity often require continuous monitoring, medication management, health information seeking, and coordination across multiple healthcare services. These needs make self-management and timely access to reliable health information particularly important in this population.

The rapid development of digital health technologies has changed the ways in which individuals access health information and interact with healthcare services. Internet-based health information, mobile health applications, online appointment systems, electronic health records, and public health service platforms have become increasingly integrated into chronic disease management (7, 8). However, the benefits of digital health technologies are not equally distributed. Older adults, particularly those with multimorbidity, may experience barriers related to limited digital access, reduced operational skills, difficulty judging the credibility of online health information, concerns about privacy and security, and anxiety or fear when using digital technologies. These barriers may further widen digital exclusion and limit the potential benefits of digital health services for older adults with complex health needs. Digital health literacy (DHL) refers to the ability to seek, understand, evaluate, and apply health information from digital sources to support health-related decision-making and self-management (9). Among older adults, DHL is not merely a technical skill; it may also reflect broader capacities involving health motivation, confidence in self-management, critical appraisal of information, technology-related psychological barriers, and available social resources. Higher DHL has been associated with better engagement in health management and improved health-related outcomes among patients with chronic conditions (10, 11). Conversely, insufficient DHL may hinder access to timely health information and reduce older adults’ ability to participate effectively in digitalized healthcare systems (12, 13).

Although previous studies have examined eHealth or digital health literacy among general older adults or patients with specific chronic diseases, evidence remains limited for community-dwelling older adults with multimorbidity. This population may face a combined burden of complex disease management, age-related functional decline, digital exclusion, technophobia, and unequal social support. In addition, few studies have simultaneously considered sociodemographic characteristics, digital access, perceived usefulness and ease of use of digital health tools, health activation, technophobia, and reciprocal social support within a single analytical framework. Clarifying these associated factors may help community healthcare providers identify older adults at greater risk of low DHL and design more targeted support strategies. Therefore, this study aimed to assess the level of DHL among community-dwelling older adults with multimorbidity and to examine sociodemographic, clinical, digital access, technology perception, and psychosocial factors associated with DHL.

## 2 Methods

### 2.1 Study design, setting, and participants

This was a community-based cross-sectional study conducted between November 2025 and February 2026 in Hefei, Anhui Province, China. Participants were recruited from three community hospitals using convenience sampling. Convenience sampling was used because a complete sampling frame of community-dwelling older adults with multimorbidity was not available in the participating communities, and data collection required the support of community hospitals and face-to-face assistance for some older participants. Although this approach was practical for recruiting the target population within the study period, it may have introduced selection bias because older adults who were more active in community health services or more willing to participate in surveys were more likely to be included.

Participants were eligible if they met all of the following criteria: (1) aged 60 years or older; (2) had lived in the study area for at least one year; (3) had multimorbidity, defined as the coexistence of two or more physician-diagnosed chronic conditions; (4) had sufficient communication and comprehension ability to complete the questionnaire independently or with investigator assistance; and (5) provided written informed consent. The list of chronic diseases was finalized based on a literature review and recommendations provided by the expert panel, together with two senior gerontological nursing experts holding full professional titles from a university and a hospital (14). The chronic conditions evaluated in this study included hypertension, diabetes, coronary heart disease, stroke, chronic obstructive pulmonary disease, asthma, hyperlipidemia, chronic kidney disease, chronic hepatitis B, and chronic gastritis. The Charlson Comorbidity Index was not used to define multimorbidity or to stratify disease severity in this study. Participants were excluded if they were experiencing an acute exacerbation of a chronic disease at the time of recruitment or had a diagnosed mental illness that prevented valid questionnaire completion.

This study was approved by the Ethics Committee of Hefei First People’s Hospital (Approval No. 2025-267-01). Written informed consent was obtained from all participants before enrollment.

### 2.2 Conceptual framework and variable domains

The measurement framework was developed to distinguish the primary outcome from explanatory constructs. DHL was treated as the primary outcome variable. Potential associated factors were organized into four domains: sociodemographic characteristics, clinical characteristics, digital access and technology perceptions, and psychosocial constructs. Sociodemographic characteristics included sex, age group, educational level, monthly income, and marital status. Clinical characteristics included duration of chronic diseases and number of chronic diseases. Digital access and technology perception variables included daily Internet use, perceived usefulness of digital health tools, and perceived ease of use. Psychosocial constructs included health activation, technophobia, and reciprocal social support. This framework was used to avoid treating the instruments as unrelated questionnaires. The Digital Health Literacy Scale was used to measure the outcome. The Health Activation Index, Technophobia Scale, and Brief Two-Way Social Support Scale were used to measure explanatory psychosocial constructs that may be associated with DHL among older adults with multimorbidity.

### 2.3 Measures

#### 2.3.1 General information questionnaire

A structured general information questionnaire was developed by the research team after reviewing relevant literature and discussing the study objectives. The questionnaire collected information on sex, age, educational level, monthly income, marital status, duration of chronic diseases, number of chronic diseases, daily Internet use, perceived usefulness of digital health tools, and perceived ease of use. Daily Internet use was categorized as <1 h, 1–3 h, or >3 h per day. Perceived usefulness of digital health tools was categorized as very useful, moderately useful, or slightly useful. Perceived ease of use was categorized as very easy, moderately easy, or difficult. These variables were included because digital access and technology perceptions may shape older adults’ willingness and ability to use digital health information and services.

#### 2.3.2 Digital health literacy scale

DHL was assessed using the Digital Health Literacy Scale developed by Liu et al. (15). This scale includes three dimensions: interaction skills, application skills, and information acquisition and evaluation skills. It consists of 15 items rated on a 5-point Likert scale. The total score ranges from 15 to 75, with higher scores indicating higher DHL. In the original validation study, the Cronbach’s alpha coefficient was 0.941. In the present study, Cronbach’s alpha was 0.98.

#### 2.3.3 Health activation index

Health activation was assessed using the Health Activation Index originally developed by Wolf et al. (16) and culturally adapted for Chinese patients with chronic diseases by Zha et al. (17). The scale assesses knowledge, self-efficacy, and behavior related to active participation in health management. It consists of 10 items rated on a 6-point Likert scale. Raw scores were transformed to a standardized score ranging from 0 to 100, with higher scores indicating higher health activation. In the present study, Cronbach’s alpha was 0.97.

#### 2.3.4 Technophobia scale

Technophobia was assessed using the Technophobia Scale originally developed by Khasawneh (18) and adapted for Chinese older adults by Sun et al. (19). The scale includes three dimensions: technology-related stress, fear of technology, and privacy and security concerns. It consists of 13 items rated on a 5-point Likert scale, with total scores ranging from 13 to 65. Higher scores indicate greater technophobia. In the present study, Cronbach’s alpha was 0.97.

#### 2.3.5 Brief two-way social support scale

Reciprocal social support was assessed using the Brief Two-Way Social Support Scale developed by Obst et al. (20) and culturally adapted for Chinese community-dwelling older adults by Cui et al. (21). The scale includes four dimensions: receiving emotional support, providing emotional support, receiving practical support, and providing practical support. It consists of 12 items rated on a 5-point Likert scale, with total scores ranging from 12 to 60. Higher scores indicate stronger reciprocal social support. In the present study, Cronbach’s alpha was 0.96. Because several scales showed Cronbach’s alpha values exceeding 0.95 in the present sample, these values were interpreted cautiously as indicators of high internal consistency that may also reflect item redundancy or highly homogeneous item content. This issue was further addressed in the Discussion and Limitations sections.

### 2.4 Data collection and quality control

Data were collected by two trained nursing postgraduate students. Before the formal survey, all investigators received standardized training on participant recruitment, informed consent procedures, questionnaire administration, item explanation, and data quality control. After written informed consent was obtained, participants completed the questionnaire on site. For participants with visual difficulty, limited literacy, or difficulty completing the questionnaire independently, trained investigators read each item aloud in a neutral manner and recorded the participant’s response without providing interpretation or guidance. Completed questionnaires were checked immediately after collection. Questionnaires with missing key variables, logical inconsistencies, withdrawal during the survey, or obvious repetitive response patterns were excluded. Repetitive response patterns were defined as identical responses across all items within a scale or across the main Likert-type scales. All data were double-entered and cross-checked by two independent researchers. Discrepancies were resolved by referring to the original questionnaires.

### 2.5 Statistical analysis

Data were analyzed using SPSS version 27.0. Categorical variables were summarized as frequencies and percentages. Because DHL and several scale scores were not normally distributed, continuous variables were summarized as median and interquartile range [M (P25, P75)]. Between-group comparisons of DHL scores were performed using the Mann–Whitney U test for two-group variables and the Kruskal–Wallis *H* test for variables with more than two groups. Spearman’s rank correlation analysis was used to examine the correlation structure among DHL, ordinal sociodemographic variables, digital access and technology perception variables, and psychosocial scale scores. Ordinal variables were coded in a directionally meaningful manner. Specifically, higher values represented older age, higher educational level, higher income, longer daily Internet use, lower perceived usefulness, or greater difficulty in using digital health tools, as appropriate. The DHL total score was treated as an approximately continuous outcome because it was calculated by summing 15 Likert-type items. Hierarchical linear regression was used to examine factors associated with DHL. Model 1 included sociodemographic and clinical variables, including sex, age group, educational level, monthly income, marital status, duration of chronic diseases, and number of chronic diseases. Model 2 additionally included digital access and technology perception variables, including daily Internet use, perceived usefulness, and perceived ease of use. Model 3 further included psychosocial variables, including health activation, technophobia, and reciprocal social support. Categorical variables were entered using dummy coding, with the lowest or most clinically relevant category as the reference group.

To examine potential multicollinearity and conceptual overlap, the correlation matrix among core variables was inspected, and variance inflation factors and tolerance values were calculated in regression models. Because several digital perception and psychosocial variables were strongly correlated, sensitivity analyses were conducted by excluding variables with potential conceptual overlap or elevated variance inflation factors one at a time. These analyses were used to assess whether the main findings were robust after removing individual variables that might contribute to construct overlap. An exploratory moderation analysis was conducted to examine whether reciprocal social support moderated the association between technophobia and DHL. Technophobia and reciprocal social support were mean-centered before creating the interaction term. The moderation model adjusted for sociodemographic characteristics, clinical characteristics, digital access and technology perception variables, and health activation. Because this analysis was exploratory, the results were presented as supplementary findings. All statistical tests were two-sided, and *p* < 0.05 was considered statistically significant.

## 3 Results

### 3.1 Participant characteristics and distribution of digital health literacy

**Figure 1:**
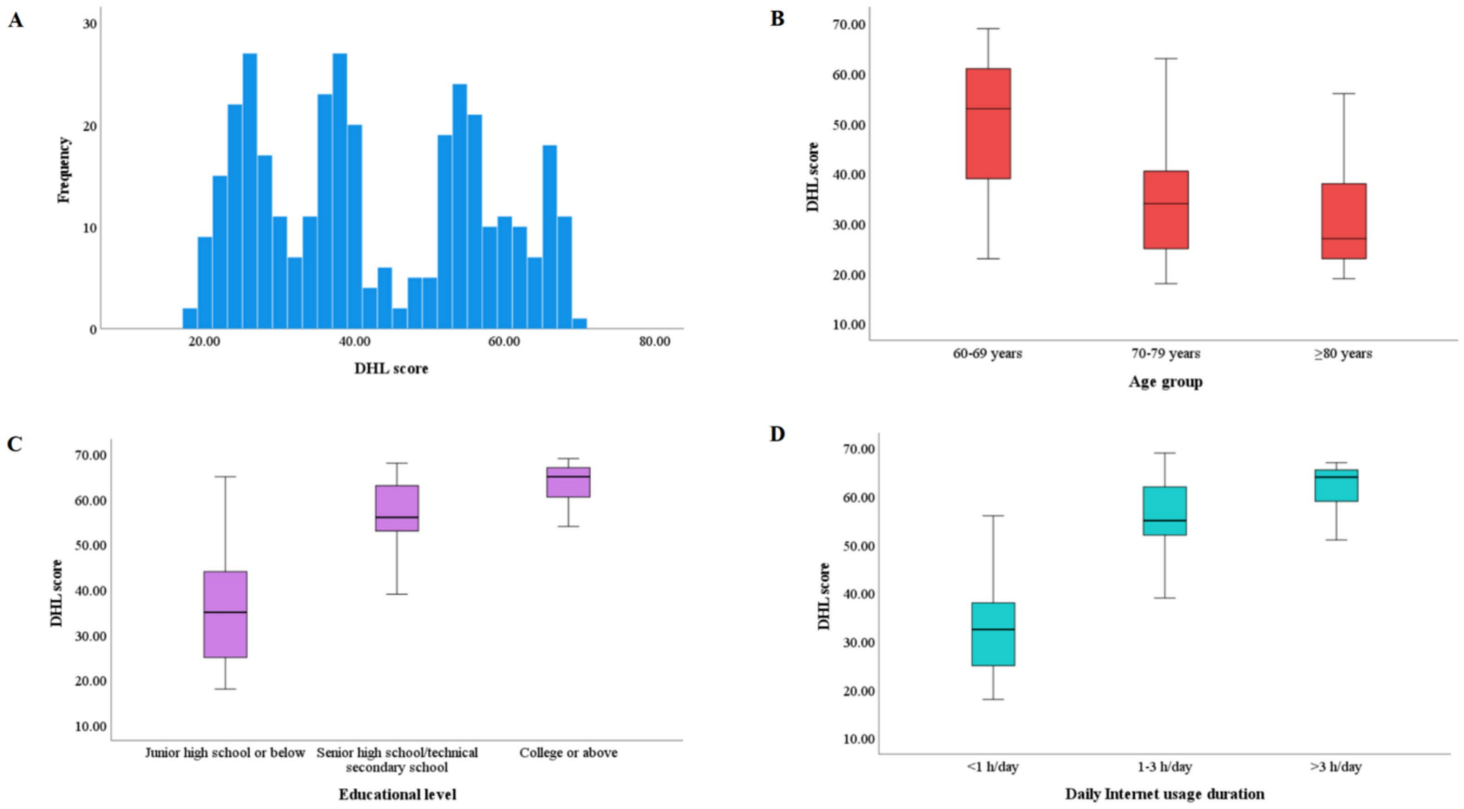
Distribution of digital health literacy scores across key participant characteristics. Panel **(A)** shows the overall distribution of DHL scores. Panels **(B)–(D)** show DHL scores by age group, educational level, and daily Internet usage duration, respectively. Boxes indicate the interquartile range, horizontal lines indicate medians, and whiskers indicate the data range excluding outliers. DHL, digital health literacy.

Of the 350 questionnaires distributed, 345 were included in the final analysis, corresponding to a valid response rate of 98.6%. Of these, 60.9% were women, 54.2% were aged 70 years or older, 74.5% had an educational level of junior high school or below, and 62.6% used the Internet for less than 1 h per day. The median DHL score was 39.00 (interquartile range [IQR], 27.00–55.00). DHL scores differed significantly by sex, age group, marital status, educational level, monthly income, daily Internet use, perceived usefulness, and perceived difficulty of using digital health tools (all *p* < 0.05). Lower DHL scores were observed among older participants and those with lower educational attainment, lower income, less frequent Internet use, less favorable perceptions of digital health information, or greater difficulty using digital health tools. DHL did not differ significantly by duration or number of chronic diseases (Table 1 and Figure 1).

**Table 1:** Participant characteristics and univariate comparisons of digital health literacy scores.

Variable	Number	Proportion (%)	Score [*M* (P25, P75), points]	Test statistic	*p* value
Sex					
Male	135	39.10	36.00 (26.00, 53.00)	−2.605^#^	0.009
Female	210	60.90	39.50 (31.75, 56.00)		
Age					
60–69 years	158	45.80	53.00 (39.00, 61.00)	97.241^*^	<0.001
70–79 years	127	36.80	34.00 (25.00, 41.00)		
≥80 years	60	17.40	27.00 (23.00, 38.00)		
Marital status					
Married	304	88.10	39.00 (29.25, 56.00)	−3.148^#^	0.002
Unmarried/Widowed/Divorced	41	11.90	30.00 (23.50, 51.50)		
Educational level					
Junior high school or below	257	74.50	35.00 (25.00, 44.00)	137.406^*^	<0.001
Senior high school/technical secondary school	61	17.70	56.00 (53.00, 63.00)		
College or above	27	7.80	65.00 (60.00, 67.00)		
Monthly average income (CNY)					
≤2,000	203	58.80	31.00 (25.00, 39.00)	−12.754^#^	<0.001
>2,000	142	41.20	56.00 (51.75, 63.00)		
Duration of chronic diseases (years)					
<5 years	101	29.30	41.00 (30.00, 56.00)	1.006^*^	0.605
5–10 years	126	36.50	38.00 (27.00, 54.25)		
>10 years	118	34.20	38.50 (26.00, 55.00)		
Number of chronic diseases (*n*)					
2	219	63.50	39.00 (28.00, 55.00)	2.192^*^	0.334
3	99	28.70	39.00 (27.00, 54.00)		
≥4	27	7.80	35.00 (25.00, 54.00)		
Daily internet usage duration (hours)					
<1	216	62.60	32.50 (25.00, 38.00)	182.792^*^	<0.001
1–3	106	30.70	55.00 (52.00, 62.00)		
>3	23	6.70	64.00 (58.00, 66.00)		
Perceived usefulness					
Very useful	71	20.60	60.00 (55.00, 65.00)	197.666^*^	<0.001
Moderately useful	167	48.40	41.00 (35.00, 54.00)		
Slightly useful	107	31.00	25.00 (23.00, 31.00)		
Perceived ease of use					
Very easy	65	18.84	62.00 (56.50, 66.00)	216.204^*^	<0.001
Moderately easy	125	36.23	51.00 (38.00, 55.00)		
Difficult	155	44.93	27.00 (24.00, 36.00)		

^#^*Z*-value for Mann–Whitney *U* test; *H-value for Kruskal–Wallis *H* test.

### 3.2 Scale scores and correlation structure

**Figure 2:**
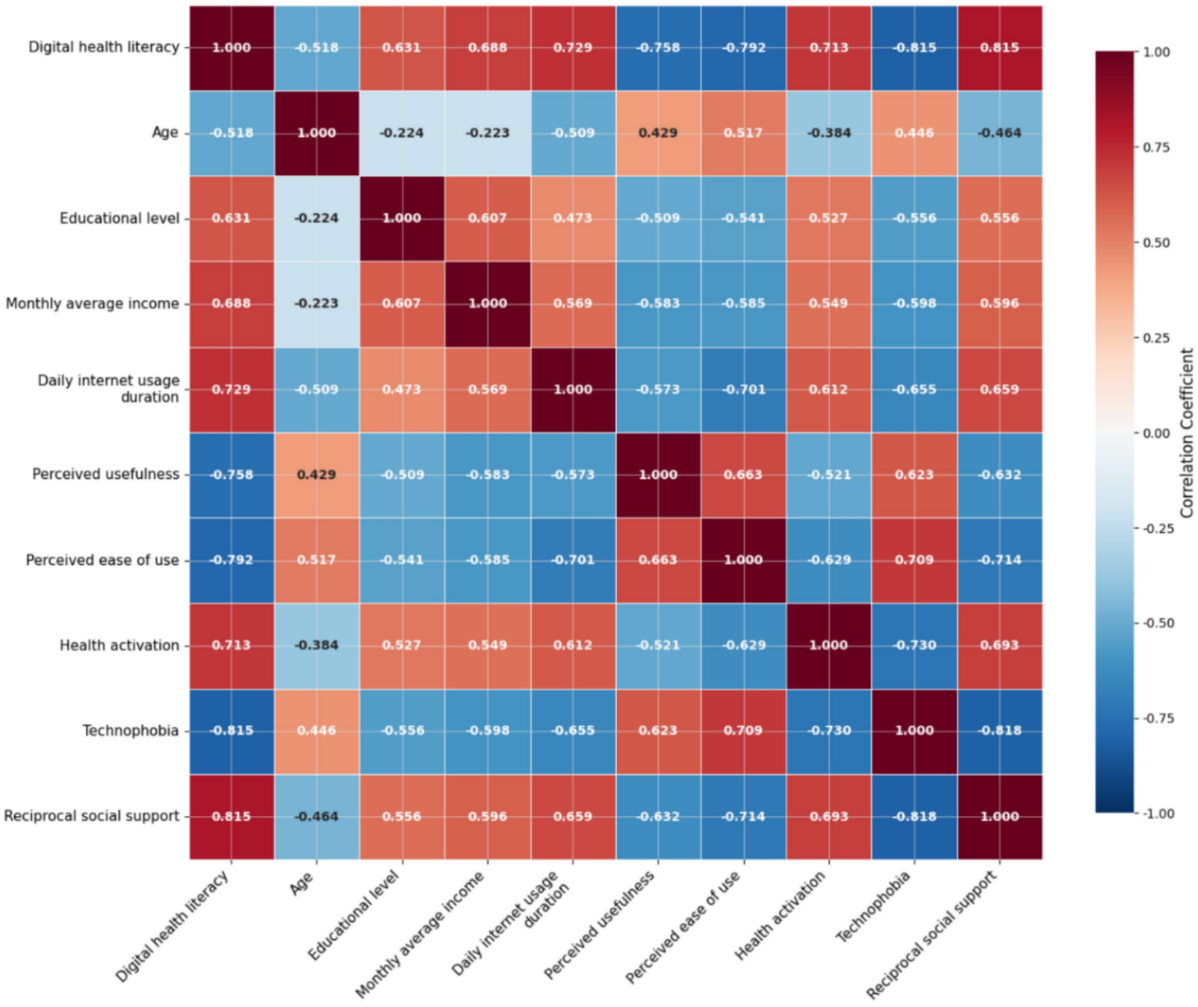
Correlation heatmap of digital health literacy and key explanatory variables. The color gradient represents correlation coefficients from −1.00 (dark blue, complete negative association) to 1.00 (dark red, complete positive association). Numerical values in each cell refer to the pairwise correlation magnitude between two variables. DHL, digital health literacy.

The median scores for health activation, technophobia, and reciprocal social support were 40.00 (IQR, 30.00–70.00), 44.00 (IQR, 27.00–52.00), and 33.00 (IQR, 29.00–45.00), respectively. Cronbach’s alpha coefficients ranged from 0.96 to 0.98. Health activation (rs = 0.713) and reciprocal social support (rs = 0.815) were positively correlated with DHL, whereas technophobia was negatively correlated with DHL (rs = −0.815; all *p* < 0.001; Table 2). Strong correlations were also observed among several explanatory constructs, particularly between technophobia and reciprocal social support (rs = −0.818), technophobia and health activation (rs = −0.730), and perceived difficulty of use and reciprocal social support (rs = −0.714; Figure 2 and Supplementary Table S1).

**Table 2:** Scale scores, internal consistency, and bivariate correlations with digital health literacy.

Construct	Role in this study	Instrument	No. of items	Possible score range	Observed score, median (P25, P75)	Cronbach’s *α*	Correlation with DHL, rs	*P* value
Digital health literacy	Outcome variable	Digital health literacy scale	15	15–75	39.00 (27.00, 55.00)	0.98	Reference	Reference
Health activation	Explanatory psychosocial variable	Health activation index	10	0–100	40.00 (30.00, 70.00)	0.97	0.713	<0.001
Technophobia	Explanatory psychosocial variable	Technophobia scale	13	13–65	44.00 (27.00, 52.00)	0.97	−0.815	<0.001
Reciprocal social support	Explanatory psychosocial variable	Brief two-way social support scale	12	12–60	33.00 (29.00, 45.00)	0.96	0.815	<0.001

### 3.3 Hierarchical linear regression models for digital health literacy

Table 3 presents the hierarchical linear regression models examining factors associated with digital health literacy. The sociodemographic and clinical model explained 70.2% of the variance in DHL (adjusted *R*^2^ = 0.692). Adding digital access and technology perception variables increased the explained variance by 13.0 percentage points (*R*^2^ = 0.832, adjusted *R*^2^ = 0.824; *P* for *R*^2^ change < 0.001). The addition of health activation, technophobia, and reciprocal social support contributed a further 5.2 percentage points, resulting in an *R*^2^ of 0.884 and an adjusted *R*^2^ of 0.877 (*P* for *R*^2^ change < 0.001).

**Table 3:** Hierarchical linear regression models for factors associated with digital health literacy.

Variable	Model 1				Model 2				Model 3			
	B (95% CI)	Beta	*t*-value	*p*-value	B (95% CI)	Beta	*t*-value	*p*-value	B (95% CI)	Beta	*t*-value	*p*-value
Constant	24.313 (18.815, 29.812)		8.699	<0.001	39.173 (33.900, 44.445)		14.616	<0.001	35.731 (27.154, 44.308)		8.196	<0.001
Sex	1.175 (−0.686, 3.036)	0.039	1.242	0.215	0.638 (−0.776, 2.053)	0.021	0.888	0.375	0.789 (−0.399, 1.978)	0.026	1.307	0.192
Age = 70–79 years	−8.257 (−10.300, −6.213)	−0.269	−7.947	<0.001	−2.120 (−3.856, −0.384)	−0.069	−2.403	0.017	−1.641 (−3.092, −0.191)	−0.054	−2.226	0.027
Age = ≥80 years	−12.833 (−15.468, −10.198)	−0.329	<0.001	<0.001	−3.381 (−5.717, −1.044)	−0.087	−2.846	0.005	−2.456 (−4.422, −0.489)	−0.063	−2.457	0.015
Marital status	0.317 (−2.505, 3.139)	0.007	0.221	0.825	0.713 (−1.445, 2.871)	0.016	0.650	0.516	0.845 (−0.982, 2.671)	0.018	0.910	0.364
Educational level = Senior high school/technical secondary school	8.174 (5.409, 10.940)	0.211	5.815	<0.001	4.603 (2.425, 6.781)	0.119	4.158	<0.001	2.291 (0.425, 4.158)	0.059	2.415	0.016
Educational level = College or above	13.368 (9.560, 17.176)	0.243	6.906	<0.001	8.328 (5.219, 11.437)	0.151	5.269	<0.001	4.967 (2.311, 7.622)	0.090	3.679	<0.001
Monthly average income (CNY)	13.518 (11.256, 15.780)	0.450	11.754	<0.001	5.644 (3.661, 7.626)	0.188	5.601	<0.001	3.549 (1.859, 5.240)	0.118	4.131	<0.001
Duration of chronic diseases = 5–10 years	−1.415 (−3.618, 0.788)	−0.046	−1.263	0.207	−0.476 (−2.156, 1.205)	−0.015	−0.557	0.578	−0.215 (−1.625, 1.194)	−0.007	−0.301	0.764
Duration of chronic diseases = >10 years	0.555 (−1.776, 2.886)	0.018	0.468	0.640	−0.174 (−1.973, 1.625)	−0.006	−0.190	0.849	0.499 (−1.022, 2.020)	0.016	0.645	0.519
Number of chronic diseases (*n*) = 3	−1.646 (−3.640, 0.348)	−0.050	−1.624	0.105	−0.343 (−1.881, 1.195)	−0.010	−0.438	0.662	0.318 (−0.979, 1.615)	0.010	0.483	0.630
Number of chronic diseases (*n*) = ≥4	−2.630 (−6.073, 0.812)	−0.048	−1.503	0.134	−1.679 (−4.304, 0.946)	−0.030	−1.258	0.209	−0.803 (−3.001, 1.395)	−0.015	−0.719	0.473
Daily internet usage duration = 1–3 h/day					6.696 (4.603, 8.789)	0.209	6.294	<0.001	3.442 (1.609, 5.275)	0.107	3.695	<0.001
Daily internet usage duration = 3 h/day					7.722 (4.102, 11.343)	0.130	4.196	<0.001	5.778 (2.722, 8.833)	0.097	3.720	<0.001
Perceived usefulness = Moderately useful					−2.786 (−5.061, −0.510)	−0.094	−2.408	0.017	−1.480 (−3.393, 0.433)	−0.050	−1.523	0.129
Perceived usefulness = Slightly useful					−9.476 (−12.287, −6.665)	−0.296	−6.632	<0.001	−6.753 (−9.144, −4.363)	−0.211	−5.559	<0.001
Perceived ease of use = Moderately easy					−3.228 (−5.721, −0.735)	−0.105	−2.547	0.011	−1.432 (−3.536, 0.673)	−0.047	−1.338	0.182
Perceived ease of use = Difficult					−8.587 (−11.672, −5.502)	−0.289	−5.476	<0.001	−3.855 (−6.545, −1.165)	−0.130	−2.819	0.005
Health activation									0.057 (0.013, 0.100)	0.084	2.554	0.011
Technophobia									−0.232 (−0.327, −0.138)	−0.205	−4.847	<0.001
Reciprocal social support									0.253 (0.142, 0.365)	0.179	4.486	<0.001
*F* statistic	71.354				95.466				123.923			
*R*^2^	0.702				0.832				0.884			
Adjusted *R*^2^	0.692				0.824				0.877			
Model *p* value	<0.001				<0.001				<0.001			

B, unstandardized coefficient; CI, confidence interval; Beta, standardized coefficient. Hierarchical entry: Model 1 included demographic covariates; Model 2 further added daily internet usage duration, perceived usefulness, perceived ease of use; Model 3 additionally incorporated health activation, technophobia, reciprocal support.

In the fully adjusted model, participants aged 70–79 years (B = −1.641, 95% CI −3.092 to −0.191) and those aged 80 years or older (B = −2.456, 95% CI −4.422 to −0.489) had lower DHL scores than those aged 60–69 years. Senior high school or technical secondary education (B = 2.291, 95% CI 0.425 to 4.158), college education or above (B = 4.967, 95% CI 2.311 to 7.622), and monthly income above 2,000 CNY (B = 3.549, 95% CI 1.859 to 5.240) were positively associated with DHL. Compared with Internet use of less than 1 h per day, use for 1–3 h (B = 3.442, 95% CI 1.609 to 5.275) and more than 3 h (B = 5.778, 95% CI 2.722 to 8.833) was associated with higher DHL. Considering digital health information only slightly useful (B = −6.753, 95% CI −9.144 to −4.363) and reporting difficulty using digital health tools (B = −3.855, 95% CI −6.545 to −1.165) were associated with lower DHL. Higher health activation (B = 0.057, 95% CI 0.013–0.100) and reciprocal social support (B = 0.253, 95% CI 0.142–0.365) were positively associated with DHL, whereas technophobia was negatively associated with DHL (B = −0.232, 95% CI −0.327 to −0.138). Sex, marital status, chronic disease duration, and number of chronic diseases were not statistically significant in the final model (Table 3). The maximum variance inflation factor was 5.929, indicating residual multicollinearity among some explanatory variables. The regression coefficients were therefore interpreted as mutually adjusted associations rather than independent effects.

### 3.4 Sensitivity and exploratory moderation analyses

After health activation, technophobia, or reciprocal social support was removed individually, the directions of the remaining psychosocial associations were unchanged. Adjusted *R*^2^ values ranged from 0.869 to 0.875, although maximum variance inflation factors remained above 5, indicating that conceptual overlap was reduced but not eliminated (Supplementary Table S2).

In the exploratory moderation analysis, the interaction between technophobia and reciprocal social support was not statistically significant (B = 0.002, 95% CI −0.004 to 0.008; *p* = 0.554), providing no evidence that reciprocal social support modified the association between technophobia and DHL (Supplementary Table S3).

## 4 Discussion

This study examined DHL within an integrated framework encompassing sociodemographic characteristics, clinical burden, digital access, technology perceptions, and psychosocial resources among community-dwelling older adults with multimorbidity. DHL was modest overall and displayed marked age, educational, income, and Internet-use gradients. In the fully adjusted model, older age, low perceived usefulness, greater difficulty using digital health tools, and technophobia were associated with lower DHL, whereas higher education, higher income, more frequent Internet use, health activation, and reciprocal social support were associated with higher DHL. The addition of digital access and technology perception variables substantially increased the variance explained by the model, with a further, smaller contribution from the psychosocial constructs. However, the high adjusted *R*^2^, strong correlations among several explanatory variables, and variance inflation factors exceeding 5 indicate that these constructs were not statistically independent. Sensitivity analyses supported the direction of the psychosocial associations but did not eliminate shared variance, and the exploratory interaction between technophobia and reciprocal social support was not significant. The principal contribution of this study is therefore not the identification of isolated “determinants,” but the demonstration that DHL in older adults with multimorbidity is embedded in an interconnected system of socioeconomic resources, digital exposure, technology-related perceptions, motivation for self-management, and available social support.

The observed sociodemographic gradients are consistent with evidence that the digital divide among older adults extends beyond access to devices and includes inequalities in skills, confidence, information appraisal, and the capacity to translate Internet access into health-related benefit (22–29). Older age may be associated with less cumulative exposure to digital technologies and with sensory, cognitive, or motor difficulties that make complex interfaces harder to navigate. Educational attainment may facilitate comprehension of medical terminology, evaluation of information quality, and transfer of existing literacy skills to digital environments. Income may influence access to reliable devices, broadband connectivity, paid services, and informal technical assistance. Nevertheless, these associations require cautious interpretation. Monthly income was dichotomized at 2,000 CNY according to the prespecified questionnaire; this threshold should not be interpreted as a validated poverty or deprivation boundary and may have obscured a more complex socioeconomic gradient. Similarly, women had slightly higher DHL in the univariate analysis, but sex was not associated with DHL after multivariable adjustment. Because women constituted 60.9% of the sample, the overall DHL distribution may partly reflect the greater willingness of older women to participate in community surveys or their more frequent contact with community health services. The convenience sampling strategy may therefore have selected participants who were more socially engaged, healthier, or more receptive to health education than the broader population of older adults with multimorbidity. This limits population-level inference despite adjustment for measured demographic characteristics.

Internet use and technology perceptions showed particularly strong associations with DHL. Technology-acceptance research indicates that older adults are more likely to engage with digital systems when the expected benefit is clear, the effort required is manageable, and assistance is readily available (30, 31). For individuals managing multiple conditions, usefulness is likely to depend on whether digital tools simplify concrete tasks—such as obtaining reliable information, monitoring health indicators, arranging appointments, communicating with clinicians, or managing medications—rather than adding procedural complexity. Difficulty of use may encompass interface design, terminology, authentication procedures, privacy concerns, and uncertainty about recovering from errors. Technophobia adds an affective barrier: fear, stress, or mistrust may discourage experimentation and reduce opportunities to develop competence (32). Importantly, the cross-sectional design cannot establish the direction of these relationships. Frequent Internet use may improve familiarity and provide opportunities to acquire digital skills, but older adults with higher DHL may also be more likely to use the Internet regularly. Likewise, people with greater digital competence may judge digital tools as more useful and easier to use. The findings should therefore be understood as evidence of mutually reinforcing relationships rather than proof that increasing Internet use alone will cause an improvement in DHL. They also argue against viewing digital difficulty as an inevitable consequence of ageing; appropriately paced instruction, age-friendly interfaces, repeated practice, and non-stigmatizing support may enable older adults to acquire digital skills (33).

Health activation and reciprocal social support remained associated with DHL after adjustment for demographic, clinical, and digital variables. Health activation captures the knowledge, confidence, and motivation required to participate in care and self-management (34–36). Older adults with greater activation may be more willing to seek information, compare sources, ask questions, and persist when digital systems are unfamiliar. Evidence from chronic disease populations indicates that activation-oriented interventions can improve behavioral, psychosocial, and selected clinical outcomes, although the extent to which these improvements translate into sustained digital competence remains uncertain (37). Reciprocal social support may contribute through practical assistance, emotional encouragement, and opportunities for observational learning. Family members, peers, and community health workers can help older adults operate devices, interpret online information, resolve technical problems, and judge whether a source is trustworthy. Research in community-dwelling Chinese older adults similarly links social capital and support with eHealth literacy, self-efficacy, and lower technophobia (29, 34). Nevertheless, the interaction between technophobia and reciprocal social support was not statistically significant. Thus, this study did not demonstrate that general social support buffered the negative association between technophobia and DHL. Support and technophobia may instead have additive associations, or a buffering effect may depend specifically on technology-related instrumental support rather than general reciprocal support. Interaction effects are also harder to detect than main effects, and the study may not have had sufficient statistical power to identify a modest moderation effect. Accordingly, the null interaction should be interpreted as absence of evidence in this sample rather than evidence that supportive relationships are irrelevant.

The unusually high explanatory power of the final model warrants particular scrutiny. An adjusted *R*^2^ of 0.877 is uncommon for a complex behavioral construct and should not be interpreted as evidence that the model has near-complete predictive validity. DHL was strongly correlated with technophobia and reciprocal social support, while perceived usefulness and difficulty of use are conceptually close to components of DHL itself. The increase in the magnitudes of psychosocial coefficients when correlated variables were removed, together with maximum VIF values that remained above 5 in the sensitivity analyses, indicates substantial shared variance. Because all measures were collected through self-report questionnaires at the same time, common method variance may have further strengthened the observed associations (38). The high Cronbach’s alpha values require similar caution. Although questionnaires with obvious repetitive response patterns were excluded during quality control, alpha coefficients of 0.96–0.98 may reflect highly homogeneous or redundant items rather than unequivocally superior reliability (39). The available data did not permit detailed item-level diagnostics, assessment of subtle straight-lining, or formal evaluation of discriminant validity. Previous reviews have also noted that many eHealth-literacy instruments for older adults have incomplete evidence regarding dimensionality, criterion validity, and clinical interpretability (40). The regression estimates should therefore be regarded as mutually adjusted associations within a correlated measurement system, not as independent causal contributions. Future studies should combine self-report scales with objective performance tasks, examine item and factor structures, and use latent-variable methods to distinguish DHL from technology anxiety, perceived usability, activation, and social resources.

The findings nonetheless have practical relevance for community-based multimorbidity care. Assessment should extend beyond whether an older person owns a smartphone or has Internet access and should consider perceived usefulness, specific usability barriers, confidence, information-appraisal ability, and access to trusted assistance. A stepped intervention could first address access and basic navigation, then build confidence through guided practice, and subsequently develop more advanced skills in evaluating and applying digital health information. Training should use clinically meaningful tasks, plain language, accessible interfaces, repetition, and opportunities for face-to-face support. Reviews suggest that structured DHL interventions can improve eHealth-literacy scores, knowledge, and self-efficacy, although the evidence remains limited by small samples and heterogeneous intervention designs (41, 42). Several study limitations should frame these implications. The cross-sectional design precludes causal inference and leaves reverse causality plausible. Convenience sampling from three community hospitals in one city, together with the female predominance, limits generalizability to men, rural residents, institutionalized or hospitalized patients, and older adults who are disconnected from community services. Multimorbidity was defined by the number of physician-diagnosed conditions; disease severity, functional impairment, specific disease combinations, and treatment complexity were not quantified. Consequently, the absence of an association between chronic disease count and DHL should not be interpreted as evidence that clinical burden is unimportant. The binary income measure may have resulted in residual socioeconomic confounding, and investigator-assisted questionnaire completion may have introduced social-desirability or interviewer effects despite standardized procedures. Longitudinal, multicenter studies using probability-based sampling, more detailed socioeconomic and clinical measures, objective digital tasks, and externally validated models are needed. Within these constraints, the findings support a broader interpretation of DHL as a socially and psychologically situated capability that requires not only technical education, but also usable digital systems, meaningful clinical value, reduced technology-related fear, and accessible human support.

## 5 Conclusion

In conclusion, DHL among community-dwelling older adults with multimorbidity was modest and was patterned by socioeconomic position, digital exposure, technology perceptions, and psychosocial resources. Older age, low perceived usefulness, difficulty using digital health tools, and technophobia were associated with lower DHL, whereas higher education, income, Internet use, health activation, and reciprocal social support were associated with higher DHL. Given the cross-sectional design, strong intercorrelations among several constructs, and residual multicollinearity, these findings should be interpreted as mutually adjusted associations rather than independent causal effects. Community health services should integrate age-friendly, task-oriented digital training with usability improvements, support for chronic disease self-management, strategies to reduce technology-related fear, and accessible assistance from family members, peers, and healthcare professionals. Longitudinal, multicenter studies using representative samples and objective assessments of digital skills are needed to clarify temporal relationships and validate these findings.

## Data Availability

The raw data supporting the conclusions of this article will be made available by the authors, without undue reservation.
