# Supplementary material for: Digital health literacy and associated factors among community-dwelling older adults with multimorbidity: a cross-sectional study
Source: Front Public Health. 2026 Jul 8;14:1841486. doi: 10.3389/fpubh.2026.1841486 (PMC13388923; doi:10.3389/fpubh.2026.1841486)
Supplement: Supplementary file 1 [file Supplementary_file_1.pdf]

**Table S1. Full correlation matrix among variables included in regression models**

| | 1 | 2 | 3 | 4 | 5 | 6 | 7 | 8 | 9 | 10 |
|---|---|---|---|---|---|---|---|---|---|---|
| 1. Digital health literacy | 1 | | | | | | | | | |
| 2. Age | -.518** | 1 | | | | | | | | |
| 3. Educational level | .631** | -.224** | 1 | | | | | | | |
| 4. Monthly average income | .688** | -.223** | .607** | 1 | | | | | | |
| 5. Daily internet usage duration | .729** | -.509** | .473** | .569** | 1 | | | | | |
| 6. Perceived usefulness | -.758** | .429** | -.509** | -.583** | -.573** | 1 | | | | |
| 7. Perceived ease of use | -.792** | .517** | -.541** | -.585** | -.701** | .663** | 1 | | | |
| 8. Health activation index | .713** | -.384** | .527** | .549** | .612** | -.521** | -.629** | 1 | | |
| 9. Technophobia | -.815** | .446** | -.556** | -.598** | -.655** | .623** | .709** | -.730** | 1 | |
| 10. Reciprocal social support | .815** | -.464** | .556** | .596** | .659** | -.632** | -.714** | .693** | -.818** | 1 |

Note: *$P$< 0.05.; **$P$ < 0.01.

**Table S2. Sensitivity analyses excluding variables with potential conceptual overlap**

| Model | Excluded variable | Health activation B (95% CI) | Technophobia B (95% CI) | Reciprocal social support B (95% CI) | Adjusted $R^2$ | Maximum VIF |
|---|---|---|---|---|---|---|
| Full model | None | 0.057 (0.013, 0.100) | -0.232 (-0.327, -0.138) | 0.253 (0.142, 0.365) | 0.877 | 5.929 |
| Model A | Technophobia | 0.095 (0.053, 0.137) | | 0.384 (0.283, 0.485) | 0.869 | 5.900 |
| Model B | Reciprocal social support | 0.070 (0.025, 0.114) | -0.335 (-0.420, -0.250) | | 0.870 | 5.787 |
| Model C | Health activation | | -0.276 (-0.365, -0.187) | 0.272 (0.161, 0.383) | 0.875 | 5.903 |

Note: Sensitivity analysis was performed by sequentially removing one predictor variable each time to assess the robustness of regression estimates against multicollinearity and conceptual overlap. Model A excluded Technophobia; Model B excluded Reciprocal social support; Model C excluded Health activation.

**Table S3. Exploratory moderation analysis of reciprocal social support on the association between technophobia and digital health literacy**

| Variables | B | SE | 95%CI | *P* value |
|---|---|---|---|---|
| Technophobia | -0.274 | 0.045 | (-0.363, -0.185) | <0.001 |
| Reciprocal social support | 0.270 | 0.057 | (0.159, 0.382) | <0.001 |
| Technophobia*Reciprocal social support | 0.002 | 0.003 | (-0.004, 0.008) | 0.554 |
